# CLARIFY: cell–cell interaction and gene regulatory network refinement from spatially resolved transcriptomics

**DOI:** 10.1093/bioinformatics/btad269

**Published:** 2023-06-30

**Authors:** Mihir Bafna, Hechen Li, Xiuwei Zhang

**Affiliations:** School of Computational Science and Engineering, College of Computing, Georgia Institute of Technology, Atlanta, GA, 30332, United States; School of Computational Science and Engineering, College of Computing, Georgia Institute of Technology, Atlanta, GA, 30332, United States; School of Computational Science and Engineering, College of Computing, Georgia Institute of Technology, Atlanta, GA, 30332, United States

## Abstract

**Motivation:**

Gene regulatory networks (GRNs) in a cell provide the tight feedback needed to synchronize cell actions. However, genes in a cell also take input from, and provide signals to other neighboring cells. These cell–cell interactions (CCIs) and the GRNs deeply influence each other. Many computational methods have been developed for GRN inference in cells. More recently, methods were proposed to infer CCIs using single cell gene expression data with or without cell spatial location information. However, in reality, the two processes do not exist in isolation and are subject to spatial constraints. Despite this rationale, no methods currently exist to infer GRNs and CCIs using the same model.

**Results:**

We propose CLARIFY, a tool that takes GRNs as input, uses them and spatially resolved gene expression data to infer CCIs, while simultaneously outputting refined cell-specific GRNs. CLARIFY uses a novel multi-level graph autoencoder, which mimics cellular networks at a higher level and cell-specific GRNs at a deeper level. We applied CLARIFY to two real spatial transcriptomic datasets, one using seqFISH and the other using MERFISH, and also tested on simulated datasets from scMultiSim. We compared the quality of predicted GRNs and CCIs with state-of-the-art baseline methods that inferred either only GRNs or only CCIs. The results show that CLARIFY consistently outperforms the baseline in terms of commonly used evaluation metrics. Our results point to the importance of co-inference of CCIs and GRNs and to the use of layered graph neural networks as an inference tool for biological networks.

**Availability and implementation:**

The source code and data is available at https://github.com/MihirBafna/CLARIFY.

## 1 Introduction

In the complex human body system, cells continually interact with one another through a series of biochemical signals. This communication helps the encompassing tissue—an ordered collection of multiple cell types—maintain its shape and function. These extracellular signaling interactions (CCIs) often occur when ligands secreted from one cell bind to receptors on another cell. Identifying these interactions is critical to understanding the role of individual cells in maintaining tissue homeostasis, while responding to their microenvironment (Rouault and Hakim 2012; Zhou *et al.* 2018). Thus, methods have been developed to elucidate these cell–cell interactions (Almet *et al.* 2021; Armingol *et al.* 2021; Dimitrov *et al.* 2022).

These methods are largely based on single-cell (sc)-RNA seq data, and unfortunately, result in the positive labeling of many false interactions. For example, a cell expressing a ligand may be deemed to interact with another cell expressing the receptor, regardless of their spatial location. In reality, the interaction can happen only if the pair is proximal as the ligand can only diffuse so far through a tissue. With the rise of spatial transcriptomics, we are now able to not only understand gene expression in a single cell, but also identify the spatial location of the cell expressing the gene (Ståhl *et al.* 2016; Wang *et al.* 2018; Eng *et al.* 2019; Rodriques *et al.* 2019). Now the methods have introduced post-processing steps to cut down on false-positive interactions by eliminating distant predicted interaction (Efremova *et al.* 2020; Garcia-Alonso *et al.* 2021). And, those that use spatial transcriptomics data from the start mainly predict cell-type level interactions (Cang and Nie 2020; Efremova *et al.* 2020; Shao *et al.* 2022).

Note that these extracellular interactions are not standalone, but occur alongside intracellular molecular interactions. Gene expressions are known to be regulated by transcription factors (TFs), which are also encoded by genes. Together they form networks called gene regulatory networks (GRNs) (Levine and Davidson 2005). Many methods have also been developed for GRN inference using gene expression data, mostly for bulk cells (Pratapa *et al.* 2020), while some infer cell-type specific GRNs (Chasman and Roy 2017; Wang *et al.* 2021). A few known methods have been created for single cell-specific GRN inference (Zhang *et al.* 2022b; Zhang and Stumpf 2023). However, to our knowledge, there are no published methods for inferring GRNs using spatial transcriptomic data.

To summarize, both CCI inference and GRN inference have been extensively researched in the last few years even at the single cell level. However, current methods view the two tasks as being essentially separate. In reality, however, intracellular signaling (through GRNs) affects extracellular signaling (CCIs) and vice versa. Extending from our previous example, when a ligand from one cell binds to a receptor on another, it will activate or repress a signal transduction pathway in the second cell, thus significantly impacting the GRN of the second cell. Similarly, the extracellular signals generated from cell 2 may, in turn, further activate or repress the communication from cell 1. Therefore, while many methods have been published for CCI inference that incorporate spatial constraints, they are still plagued with a high number of false positives, as downstream gene regulatory information is not incorporated. Similarly, with GRN inference, there is a need to infer spatial context aware and cell-specific GRNs. Here, we make the reasonable assumption that the closer two cells are in spatial proximity, not only are they more likely to engage in a CCI, but also their GRNs should be more similar as they will engage in similar regulatory actions. The cells that are spatially close AND of the same type shall have the most similar GRNs, for the aforementioned reasons. Using this idea, we propose the first method for a joint refinement of spatially-aware CCI and GRNs.

While it is logical to motivate the need for joint inference of extracellular and intracellular interactions, developing computational methods for simulating and inferring these complex signaling pathways remains a challenging task. Our method relies on first viewing this entire network of interactions as a multi-level knowledge graph incorporating information from the cell-level and the gene-level. Our method then utilizes graph neural networks (GNNs) to embed both the cell-level and the gene-level information together into a robust latent representation. GNN based methods have become largely ubiquitous in the computational biology domain (Yuan and Bar-Joseph 2020; Li and Yang 2022) and in biomedicine/drug discovery as well (Li *et al.* 2022b; Zeng *et al.* 2022) largely because of their ability to take advantage of contextual information (Tie and Pe 2022). They have been used in myriad situations where spatial context was important and have recently made breakthroughs in biological findings (Wu *et al.* 2022; Zhang *et al.* 2022a). This motivates GNNs as a fitting candidate for our task to learn our multi-level knowledge graph.

We propose CLARIFY, a multi-level graph autoencoder (GAE) that refines intracellular and extracellular interaction networks by utilizing the spatial organization of single cells given by spatial transcriptomics data. CLARIFY takes as input spatial transcriptomics data and produces cell-level, gene-level, and combined embeddings that encapsulate the single cell gene expression, spatial context, and gene regulatory information to aid in the refinement of extracellular/intracellular interactions. We test CLARIFY on two real datasets and one simulated dataset. For the task of CCI reconstruction, we compare the performance of CLARIFY with the only other existing semi-supervised learning method for this task: DeepLinc (Li and Yang 2022). Additionally, on simulated data, where ground truth GRNs and cell-type CCIs are available, we compare the CCI inference with SpaOTsc (Cang and Nie 2020), and compare the GRN inference with Genie3 (Huynh-Thu *et al.* 2010). We show that CLARIFY outperforms existing methods in both cell-level and gene-level tasks, while tackling the problem jointly unlike the baselines. This, along with our multiple spatial enrichment experiments confirm that CLARIFY is able to refine both the cell-level and the gene-level regulatory interaction networks, clarifying the true spatially constrained dynamic of the tissue.

## 2 Materials and methods

Here, we describe our multi-level graph autoencoder (GAE) approach, starting with the input knowledge graph construction, then graph neural network inference, and finally the training objective.

### 2.1 Multi-level graph construction

**Figure 1.**
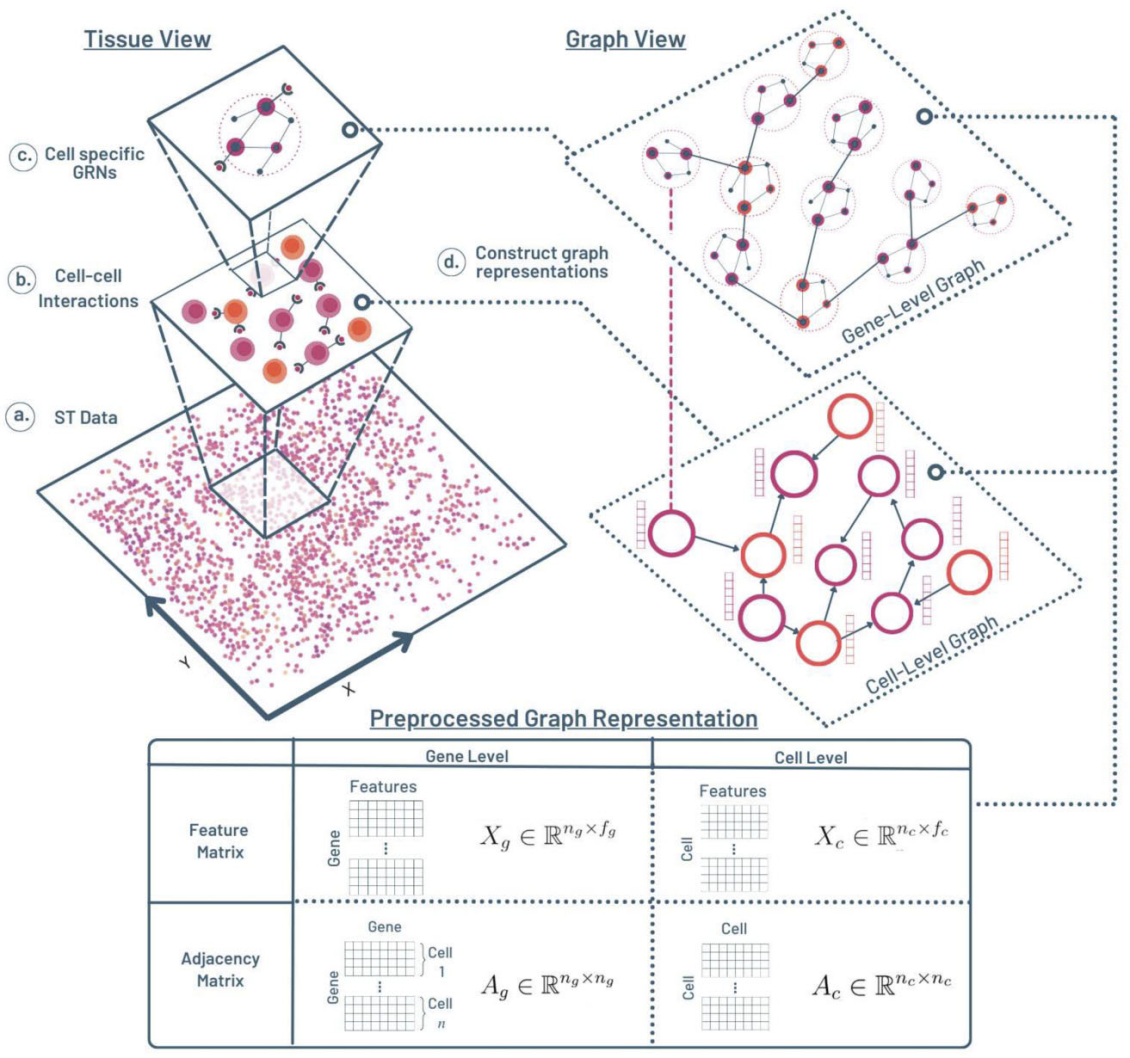
Multi-level graph construction. The left column represents the interactions occurring in the tissue and the right column represents the multi-level graph view that models the interactions. (a) It depicts view of the starting spatial transcriptomics data, (b) it shows the view of the spatially constrained cell–cell interactions (usually mediated by ligand–receptor connections), and (c) views the within-cell gene regulatory interactions. The views from (b) and (c) are constructed into separate cell-level and gene-level graphs respectively, shown in (d) and the right column.

To address the shortcomings of current methods in extra/intra-cellular interaction prediction, our multi-level construction can be broken into two main views: cell-level and gene-level. The goal of the cell-level graph is to encode the notion of spatial constraints and the gene-level graph provides the downstream gene regulatory information. For simplicity, we denote every cell-level element with subscript ‘c’ and gene-level element with subscript ‘g’. For this section, refer to Fig. 1.

**Figure 2.**
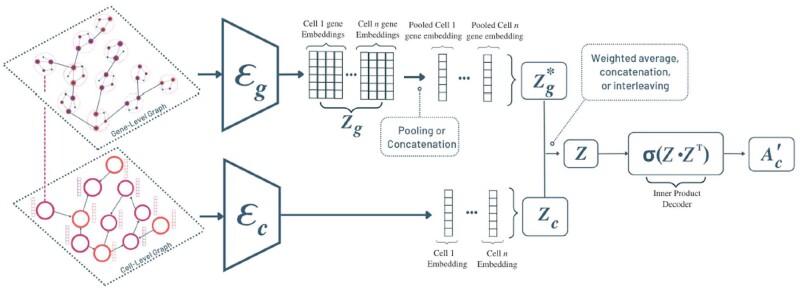
Illustration of the proposed multi-level Graph autoencoder architecture, CLARIFY.

#### 2.1.1 Cell-level graph

At the cell level, we view each single cell as a vertex in our graph. To utilize the spatial component of our data, we connect edges between cell vertices based on spatial proximity. If no ground truth interactions are available, we used a *k*-NN algorithm on the spatial transcriptomics data to determine edges. We denote the adjacency matrix describing the vertices and edges as $A_{c}\in R^{n_{c}\times n_{c}}$, where $n_{c}$ is the number of cells. $A_{i,j}=1$ if there exists an edge connecting cell *i* and cell *j*; $A_{i,j}=0$ otherwise.

Finally, each cell (vertex) in our graph will have an attributed feature vector based on the single cell expression values (each row of the ST data). This can be organized into a feature matrix $X_{c}\in R^{n_{c}\times f_{c}}$, where $f_{c}$ stands for the number of features (genes) per cell.

Together, adjacency matrix $A_{c}$ and feature matrix $X_{c}$ make up our cell-level proximity graph $G_{c}$, which will be used as one part of the training input to our model. In essence, the purpose of this cell-level graph construction is to introduce, to our model, the notion of cells that have the capacity to interact based on their spatial location in the tissue.

#### 2.1.2 Gene-level graph

At the gene level, we essentially take the cell level graph one step further, by viewing each single cell as a subgraph of its underlying cell-specific gene regulatory network (GRN). To do this, we must first infer baseline cell-specific gene regulatory networks with the CeSpGRN method (Zhang *et al.* 2022b). Note that cell-type level GRN inferences can also be utilized, but cell-specific methods encourage more cell–cell variability. As the first part of the gene-level preprocessing, we take in the input cell-level feature matrix $X_{c}\in R^{n_{c}\times f_{c}}$ defined in the previous subsection. CeSpGRN then infers and outputs a gene regulatory network for each single cell, as a list of adjacency matrices where each vertex in a single adjacency matrix represents a gene—though of the same name—which belongs to a specific cell. The gene adjacency matrix is firstly constructed by stacking the cell-specific GRN adjacency matrices diagonally, resulting in a block diagonal matrix $A_{g}\in R^{n_{g}\times n_{g}}$, where $n_{g}$ is the number of total genes, i.e. each gene in each cell corresponds to one row or column. Note that for each cell *i* in the cell-level graph $G_{c}$, there exists a corresponding GRN component in the gene-level graph $G_{g}$, which is represented by a block along the diagonal in $A_{g}$ and denoted by the pink dotted line (Fig. 1d between the cell/GRN pair across the two graphs).

We then augment the gene-level graph with inter-cellular edges by translating the proximity edges of $G_{c}$ to the GRN components of $G_{g}$.To do this, we first must understand which genes of cells have the capacity to interact with genes of neighboring cells. These cell–cell interactions (CCIs) are primarily observed by the genes corresponding to ligands and receptors. Using a standard ligand–receptor (LR) database (Shao *et al.* 2021), we identify LR genes in every GRN. The LR edges are then constructed in the following manner: given cell *i* and cell *j*, if they share an edge in $G_{c}$ (meaning they are spatially proximal), we construct an edge between every LR gene in GRN *i* and GRN *j* in $G_{g}$. That is, $A_{g_{u,v}}=1$ if *u* is in cell *i* and *v* is in cell *j*, and (u,v) is a gene pair present in the LR database. The adjacency matrix of $A_{g}$ will have intracellular (GRN) edges on the block diagonal and extracellular (CCI-LR) edges off the block diagonal.

For proper graph autoencoding, we establish an initial set of features $R^{f_{g}}$ for each vertex (gene) in our graph by using the Node2Vec method (Grover and Leskovec 2016), where each vector represents an embedding of the corresponding vertex’s local network neighborhood. The feature vectors can be grouped into a matrix $X_{g}\in R^{n_{g}\times f_{g}}$ format analogous to the cell features but differing in dimension.

The adjacency and feature matrix complete our gene-level graph construction, which can essentially be thought of as a graph of GRN subgraphs. With this gene-level graph, we effectively provide our model the knowledge of each cell’s underlying gene regulatory network which models the downstream effect of an extracellular interactions.

### 2.2 Multi-level graph autoencoder framework

#### 2.2.1 Overview

CLARIFY has four inputs: the features and binary adjacency matrices from both cell-level and gene-level graphs ($X_{c},A_{c},X_{g},A_{g}$). CLARIFY makes use of two parallel Graph Neural Network encoders (see Fig 2.) for both the cell and the gene level graphs $E_{c}(\cdot )$ and $E_{g}(\cdot )$. Each encoder embeds the respective cell or gene features into latent representations. These separate latent representations are then aggregated (either concatenation or averaging), to integrate learned information from both levels. This combined latent variable is then decoded (inner-product) into a reconstructed cell-level adjacency matrix. The model is then optimized on reconstruction ability of the cell-level adjacency, but also penalized for harsh changes in intracellular gene interactions.

#### 2.2.2 GCN layer

For the encoding layers of CLARIFY, we utilize Graph Convolutional Networks (GCNs), which is a widely used GNN architecture that have become omnipresent in the computational biology world.

Built upon message passing neural networks, a GCN can be deconstructed into a series of message passing and aggregation steps. This can be thought of as a function Z=f(X,A) that takes a graph’s vertex features *X* and adjacency *A* and uses the edges to pass messages between neighboring vertices to embed the vertex features into a more effective representation *Z*. In this way, the development of novel GCN layers is essentially a tweaking of the function f(⋅), i.e. the steps taken in message passing and aggregation. Note that we can stack these layers analogously to standard convolutional neural networks. For our model, we use stacked graph convolutional layers which has the following message-passing rule proposed by (Kipf and Welling 2016):


(1) $$Z^{\left(l+1\right)}=\sigma (\underset{\mathrm{normalization}}{\underbrace{{\tilde{D}}^{\frac{1}{2}}\tilde{A}{\tilde{D}}^{-\frac{1}{2}}}}Z^{\left(l\right)}W^{\left(l\right)}),\;\;\;\;Z^{\left(0\right)}=X.$$

At GCN layer 0, $Z^{\left(0\right)}$ is the initial input node features *X*. The graph’s input adjacency matrix is symmetrically normalized shown by the normalization step in (1). Note that $\tilde{A}=A+I_{n}$ and $\tilde{D}$ is the degree matrix of $\tilde{A}$. At each layer *l*, there is a learnable weight parameter $W^{\left(l\right)}$.

#### 2.2.3 Cell/gene level encoders

To adapt this standard GAE to our task on a graph with multiple levels, we utilize two parallel Graph Encoders–one for each level. Both graph encoders use GCN layers to embed the vertex features of their respective level as denoted below.


(2) $$Z_{c}=E_{c}(X_{c},A_{c})\;\;\;\;\;\;\;\;\;\;Z_{g}=E_{g}(X_{g},A_{g}).$$

Note that $Z_{c}\in R^{n_{c}\times d}$ and $Z_{g}\in R^{n_{g}\times d}$, where *d* is the dimension of the latent embedding space. Each row in $Z_{c}$ is the latent representation of the cell (vertex) in $G_{c}$. Each row in $Z_{g}$ is the latent representation of a gene belonging to a single cell’s GRN. We aggregate each GRN’s gene representations together into one gene-level cell embedding such that the updated matrix is of the form $Z_{g}^{*}\in R^{n_{c}\times d}$. Formally, for the *k* genes in cell *i*,


(3) $$\begin{array}{cc} Z_{g}^{*}\left[i\right]=\frac{1}{k}\sum_{j=1}^{k}\text{GRN}\left[i\right]\left[j\right] & \;\;\text{or}\;Z_{g}^{*}\left[i\right]=\overset{k}{\underset{j=1}{⊕}}\text{GRN}\left[i\right]\left[j\right] \end{array}.$$

As noted, either pooling or concatenating (written as direct sum ⊕ notation) can be used for this step of aggregation. Essentially, this step aggregates the gene-level embeddings by the cells to which they belong, effectively creating a GRN based cell-level embedding. We then integrate the information learned in the original cell level embeddings and the new (GRN) cell level embeddings, by concatenating the two matrices:


(4) $$Z=\left[\begin{array}{cc} Z_{c} & Z_{g}^{*} \end{array}\right].$$

This resulting embedding encapsulates the single cell gene expression, spatial context, and downstream gene regulatory information.

#### 2.2.4 Cell/gene level decoders

For both the cell-level and the gene-level tasks, graph reconstruction is done by the use of inner-product decoders. The inner product decoder for the cell-level makes use of the combined embedding *Z* and is defined as such:


(5) $$A_{c}^{\prime }=D_{c}(Z)=\sigma (ZZ^{⊤}),\;\;\;\;\;\;\;\;A_{c}^{\prime }\in R^{n_{c}\times n_{c}}.$$

The gene-level decoder on the other hand carries out the gene-level graph reconstruction using only the gene-level embeddings:


(6) $$A_{g}^{\prime }=D_{g}(Z_{g})=\sigma (Z_{g}Z_{g}^{⊤}),\;\;\;\;\;\;\;A_{g}^{\prime }\in R^{n_{g}\times n_{g}}.$$

The inner product decoders compute the inner product (cosine similarity score) between each pair of embeddings. Each cosine similarity score is an entry in the resulting matrix, which represents how likely an edge exists between the two candidate vertices. The sigmoid function is then applied to transform the cosine similarity matrix into probabilities that represent the existence likelihood of an edge. These, in essence, are the reconstructed values of the adjacency matrix.

### 2.3 Training objective

CLARIFY is optimized on two tasks, the first of which is its ability to reconstruct the spatial proximity edges defined by the cell level adjacency matrix $A_{c}$. For this, we utilize binary cross entropy (BCE) reconstruction loss. Note that each *i*, *j* entry of $A_{c}$ represents the ground truth label for the existence of a proximity edge between cell *i* and cell *j*. And, each *i*, *j* entry of $A_{c}^{\prime }$ represents CLARIFY’s predicted probability score for that same edge. Thus, the BCE loss is defined as such:


(7) $$\begin{array}{c} L_{c}\left(A_{c},A_{c}^{\prime }\right)=-\frac{1}{n_{c}^{2}}\sum_{i=1}^{n_{c}}\sum_{j=1}^{n_{c}}A_{c}\left[i\right]\left[j\right]\;\text{log}\left(A_{c}^{\prime }\left[i\right]\left[j\right]\right)\;+(1-A_{c}\left[i\right]\left[j\right])\;\text{log}(1-A_{c}^{\prime }\left[i\right]\left[j\right]) \end{array}.$$

As the model trains, the updated weights will drastically change each of the gene feature vectors in the gene-level graph. In order to reduce the effect of this message propagation and the cell-specific GRN information, we include a secondary loss term that ensures that the edges in each cell-specific GRN are not changed too drastically, but rather just enough to be spatially refined. Recall that the intracellular (GRN) edges are located on the block diagonal of $A_{g}$. Thus, for $L_{g}$, we use mean squared error loss between the block diagonal entries of $A_{g}$ and reconstructed $A_{g}^{\prime }$. Each block is of dimension $R^{g\times g}$, where *g* is the number of genes per cell. Formally, we let the block diagonal entries of a $A_{g}$ be defined as such:


(8) $$\begin{array}{cc} \text{mask}=\overset{n_{c}}{\underset{i=0}{⊕}}1 & \text{where}\;1\in R^{g\times g},\;\text{mask}\in R^{n_{g}\times n_{g}} \end{array}.$$

(9) $$A_{g\text{block}}=A_{g}⊙\text{mask}.$$

In other words, the mask is a matrix with 1 s in the g×g blocks along the diagonal. The entries of $A_{g}$ are then masked by element-wise multiplication ⊙. The same is done for $A_{g}^{\prime }$. Finally, the loss of the block diagonal entries is:


(10) $$L_{g}=\text{MSE}(A_{g\text{block}},A_{g\text{block}}^{\prime }).$$

We combine these losses in a weighted sum as follows. $\lambda_{i}$ are hyperparameters defined by the user depending on whether the preservation of GRN information or spatial refinement is more important. As default, they are both set to 1. The total loss is defined below:


(11) $$L=\lambda_{c}L_{c}+\lambda_{g}L_{g}.$$

## 3 Results

We evaluated CLARIFY in a series of experiments, broken up into two main components: cell-level and gene-level. Recall that CLARIFY jointly refines both cell-level (CCI) and gene-level interactions (GRN), and it is the only known method to do so. Typically, however, these problems were viewed as distinct, and independent methods were devised to solve either problem. Therefore, we evaluate CLARIFY performance separately against existing methods in each domain.

### 3.1 Experimental design

#### 3.1.1 Datasets

Due to the lack of data at single cell resolution for spatial transcriptomics, there are only a handful of datasets to be utilized. And, most of them are not extensively studied, so there are no known ground truth interactions for those real datasets. For each task, we evaluated CLARIFY and existing methods on two real spatial transcriptomics datasets and one simulated dataset. We considered two published datasets on mice. The first dataset was acquired from the mouse visual cortex using seqFISH technology (Lubeck *et al.* 2014). The data captures transcript expression from 125 genes in 1597 single cells, along with the spatial location of the expressed transcripts. The second dataset was a slice from the mouse hypothalamus using the MERFISH technology (Moffitt *et al.* 2018), which sampled 160 genes in 2000 single cells. Data from both sets was preprocessed using a standard approach (log transform over counts), also used by other tools like DeepLinc.

We also generated simulated data with scMultiSim (Li *et al.* 2022a). scMultiSim generates single cell gene expression data from multiple cell types as well as cell locations. The gene expression data is driven by the ground truth GRNs, CCIs, and cell-type structures.

#### 3.1.2 Evaluation metrics

To evaluate CLARIFY, we use two commonly applied metrics. The first is a precision-recall based framework, specifically the Average Precision (AP) score, which calculates the weighted mean of precisions achieved at each threshold. The weights are defined by the increase in recall from the previous threshold. Note, that the AP score is robust to datasets that are highly skewed as it does not use linear interpolation. Secondly, we utilize the area under the receiver operating characteristic (AUROC), where the ROC curve measures the True Positive Rate (TPR) versus False-Positive Rate (FPR) at different decision thresholds. We used the scikit-learn implementations of these methods (https://scikit-learn.org/stable/).

Each of the experiments defined in were designed to assess the main capabilities of CLARIFY on these datasets: reconstruction of the cell/gene interaction networks, and spatial refinement of the said networks.

### 3.2 Cell-level experiments

To the best of our knowledge, we have identified only one method (DeepLinc) that is aimed at cell interaction landscape reconstruction. There are indeed other CCI methods, however, most of them are at the cell-type level, and do not seek to reconstruct and impute spatially refined edges as DeepLinc and CLARIFY. Thus, our cell-level evaluations are mainly compared to DeepLinc. DeepLinc is similar in that it is a Variational Graph Autoencoder for CCI reconstruction, but it does not incorporate downstream gene regulatory information, nor does it consider the joint problem of CCI and GRN refinement. Therefore, we evaluated CLARIFY against it for only cell–cell interactions, but not gene–gene interactions. For the CCI reconstruction, we used the DeepLinc methodology of evaluation to provide a fair comparison.

#### 3.2.1 CLARIFY outperforms related methods for cell–cell interaction network reconstruction

For the task of CCI reconstruction, we first need to define a set of ground truth interactions as the real datasets do not have any. Following the same procedure described with DeepLinc and our cell-level graph construction, we constructed cell–cell adjacency matrices for each of the real datasets by using the *k* nearest neighbor (*k*NN) algorithm to find the *k* closest neighbors in Euclidean distance (using the spatial coordinates) for a cell. This follows the same assumption in DeepLinc, that in a 2D tissue, each cell could be locally interacting with k≥3 other cells. As noted in the methods section, this cell-level adjacency matrix $A_{c}$ was used as the set of ground truth interactions for CLARIFY to reconstruct.

**Figure 3.**
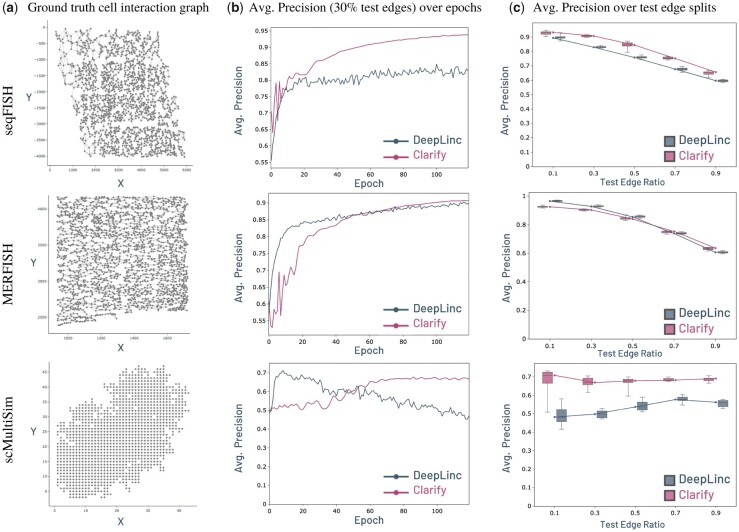
Cell-level experiment performance. (a) depiction of the spatial transcriptomics datasets with ground truth cell interaction edges. (b) CLARIFY versus DeepLinc training Average Precision over epochs. (c) CLARIFY versus DeepLinc Average Precision performance over various train/test splits denoted by the % of test edges.

To construct the training and testing split, we randomly selected 70% of edges for CLARIFY to train on and the remaining 30% were masked out and utilized for testing/evaluation. These edges are denoted as the positive set. In each training and test set, we also add randomly sampled negative edges in a 1:1 ratio with the positive edges. To assess reconstruction performance, we measured the AP and AUROC in reconstructing the test set edges over training epochs and compared them to DeepLinc’s performance. See Fig. 3b and Supplementary Fig. S4b. CLARIFY significantly outperformed DeepLinc on the seqFISH and scMultiSim datasets, while the two methods achieved comparable results on the MERFISH dataset. These results strongly suggest that CLARIFY was able to properly incorporate not only spatial information and single cell gene expression, but also the downstream network of regulating genes as part of the cell-level embeddings, and that directly influenced its performance in reconstructing cell–cell interactions.

Next, to assess CLARIFY’s robustness to different edge partitions, we also evaluated the model across all datasets while varying the size of the number of test edges. DeepLinc noted that their model was mainly trained on a split of 10% test edges leaving 90% of training. But, such a small test size may not be enough for a reconstructability task. Thus, across all datasets, we measured the AP and AUROC of test edge reconstruction over different splits ranging from 10% to 90% test edges. This was repeated 5 times for epochs 100, 110, 120 (total 15 per split) to generate the boxplots. Once again, CLARIFY outperformed DeepLinc across all splits for the seqFISH and scMultiSim datasets while gaining comparable performance for the MERFISH dataset (Fig. 3c and Supplementary Table S1), indicating robustness in maintaining performance even when training on less data. Note that for the scMultiSim simulated data, the ground truth cell interaction graph is very sparse (Fig. 3a). This contributes to the unorthodox training curves, as due to the low number of edges, each split of test edges may contain high variability, leading to slightly skewed performance for both models.

To evaluate CLARIFY’s tolerance to noisy data, we perturbed the input training graph with false-positive and false-negative edges. For false-positive edges, in the input training graph of known ligand–receptor edges, we add fake edges at rates from 0.1 to 0.5 times the original number of edges. Similarly, for the false-negative edges, we remove edges from the training set at rates from 0.1 to 0.5. We then train CLARIFY on these noisy inputs and evaluate its Average Precision score on the test set of edges for each of the cases and compare them to DeepLinc (Supplementary Fig. S2).

Lastly, for the scMultiSim simulated dataset, we obtained a cell-type CCI ground truth. As a baseline, we utilized a representative tool for cell-type level interaction prediction from spatial transcriptomics, known as SpaOTsc (Cang and Nie 2020). For each of the cell-type pairs that SpaOTsc deemed significant, we maintained in a set. We then constructed a SpaOTsc cell level adjacency matrix $R^{n_{c}\times n_{c}}$, where every *i*, *j* entry was set to 1 if cell *i*’s type and cell *j*’s type is a cell-type pair in the aforementioned set. We followed the same procedure to construct the ground truth adjacency matrix for scMultiSim and then compared CLARIFY’s reconstructed adjacency matrix with SpaOTsc’s adjacency matrix, by measuring the AP and AUROC score. Note, we also provided a baseline based on randomly permuting the scMultiSim ground truth matrix (maintaining the number of ones) 100 times and calculating the average AP and AUROC score with the normal scMultiSim ground truth. This was to provide a random baseline, to give reference for the performance of other methods. The final results are formulated in Table 1.

**Table 1.** Performance of methods on predicting scMultiSim simulated cell-type level interactions.

Method	AUROC	Avg. precision (AP)
CLARIFY	**0.710817**	**0.696506**
SpaOTsc	0.5	0.501513
Baseline (random)	0.500669	0.003024

Bold values indicate highest scoring.

**Table 2.** Correlations between distances of cell embeddings and spatial distances.

		Initial	CLARIFY
		Cell features	Cell embeddings
seqFISH	Entire matrix	0.101927	**0.22118**
	Block diagonal	0.252199	**0.69567**
MERFISH	Entire matrix	0.07148	**0.33426**
	Block diagonal	0.20234	**0.62496**

Bold values indicate highest scoring.

**Table 3.** Spearman rank correlation between spatial location and gene–gene adjacency matrices before and after CLARIFY refinement.

	Type			CLARIFY	
		Initial		Gene adjacency	
		Euclidean	Frobenius	Euclidean	Frobenius
seqFISH	Entire matrix	−0.000234	−0.005458	**0.107278**	**0.076636**
	Block diagonal	0.101489	−0.068645	**0.252312**	**0.207894**
MERFISH	Entire matrix	0.020761	**0.033948**	**0.025857**	0.013762
	Block diagonal	0.108249	0.094082	**0.181197**	**0.149367**

Bold values indicate highest scoring.

It is worth mentioning that SpaOTsc does not require any labeled data for training, while DeepLinc and CLARIFY both split the interactions into training and testing sets. The large improvement of CLARIFY over SpaOTsc and that SpaOTsc performance is close to random indicate that supervision can significantly improve the accuracy of this task.

#### 3.2.2 CLARIFY latent cell embeddings indicate valid spatial refinement and preserve spatial domains

After establishing CLARIFY’s reconstruction performance, we then assessed its ability to embed the input cell features (normalized counts) to latent representations that better contextualize the spatial distribution of cells in the tissue. These experiments help validate the claim that CLARIFY’s cell embeddings are spatially refined.

**Figure 4.**
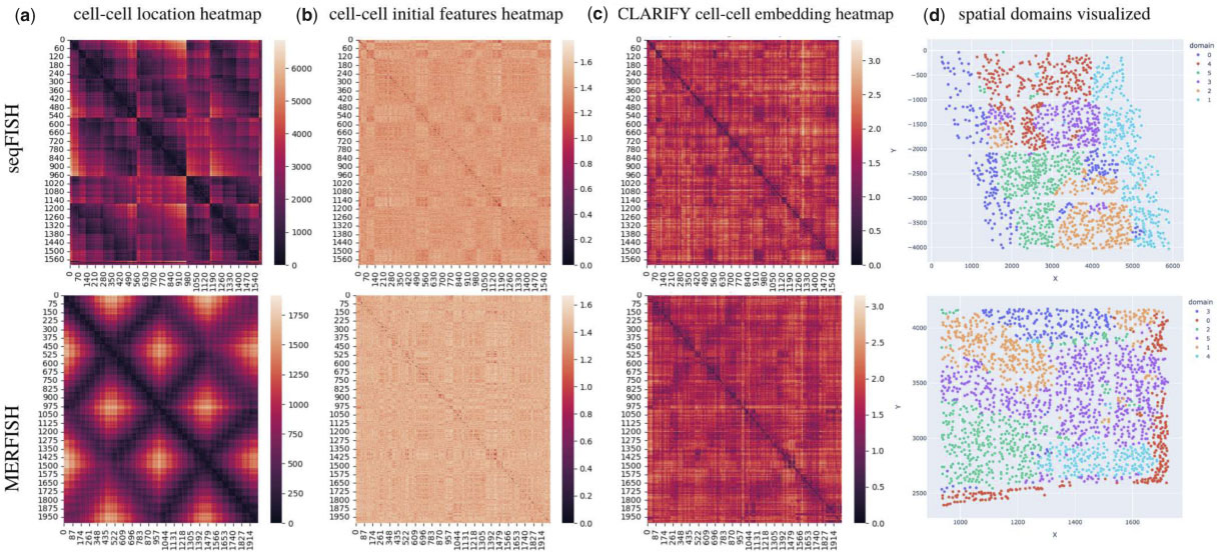
Visualization of cell level spatial refinement. (a) pairwise cell–cell heatmap based, (b) heatmap between initial cell features, (c) heatmap between CLARIFY refined cell embeddings, (d) visualization of unsupervised clusters organizing into spatial domains in the ST slice.

To provide context, we first visualize pairwise Euclidean distances between cells in Fig. 4a. In this $n_{c}\times n_{c}$ matrix, entry *i*, *j* represents the distance between cell *i* and cell *j* using the ST data coordinates. It represents the distribution of spatially located cells. We generate a representation of cell–cell similarity using both the cell’s initial features (Fig. 4b) and the cell’s latent representation produced by CLARIFY (Fig. 4c). In both cases, the entry at *i*, *j* represents the Euclidean distance between cell *i* and cell *j*’s initial feature vector or latent representation, respectively. We can see that the heatmap of the CLARIFY latent representations is visually more similar to the location distribution. For example, in Fig. 4c, the block diagonal entries (cell–cell neighborhoods) are darker (closer) similar to (Fig. 4a). In contrast, the initial feature distribution appears to be nearly uniformly distributed, and every pairwise comparison is given a similarly high Euclidean distance (indicating features are equally distant and diverse). In comparison, we note that the CLARIFY latent representations have an underlying structure, but they are not completely identical to the location distribution, which is important, as spatial location is not the only information that the embeddings encapsulate. Rather, the embeddings represent spatial location combined with gene expression, gene regulatory network information, and cell–cell interaction information.

To quantify this result, we computed the Spearman correlation between the location and the cell embedding heatmap, and as a baseline, between the location and the initial features heatmap (see Table 2). Since the entire matrix is quite large and represents sparse distal interactions, we provide the Spearman correlation between the block diagonal entries of both matrices as well. These entries represent the cell–cell neighborhoods (cells that are close together spatially shown in the location heatmap), and thus are more likely to be spatially refined. Thus, we compute this statistic for both real datasets in two scenarios: using the entire matrix and over the block diagonal entries. The results are shown in the table below. We note that the *P*-value of the Spearman correlation was highly significant in every single case (*P*-value <2e-308) because of the large number of data-points.

Across both datasets, we saw a significant improvement in the correlation when comparing the CLARIFY latent representation distribution to the location distribution, with a 2–4× increase in Spearman correlation. When using the entire matrix as comparison, there was a moderately positive correlation (0.22, 0.33), which is still interesting because the matrices represent both sparse and distal interactions. However, when using the block diagonal entries of the matrix, representing the cell–cell neighborhoods in the tissue, there was a strong positive correlation (0.696, 0.625) compared to the initial features (0.25,0.2).

As a final proof of concept, for both datasets, we clustered the cell latent representations using the k-Means algorithm (k = 6), similar to the analysis in DeepLinc. Each of the six clusters was defined as spatial domains (0 through 5) and then mapped back to each single cell and plotted (Fig. 4d). This provides another visual confirmation that even with unsupervised clustering of the embeddings, CLARIFY latent representations are clearly spatially organized into separate domains in the tissue.

All of these results strongly indicate that CLARIFY representations are spatially correlated, thus validating CLARIFY’s ability to spatially refine the single cell features.

### 3.3 Gene-level experiments

#### 3.3.1 CLARIFY cell-specific GRNs outperform existing cell-type inference methods

Currently, there are few methods that infer cell-specific GRNs (a main one is CeSpGRN, which is used for our initial graph construction). However, there are a number of cell-type GRN inference methods. The most notably benchmarked is the Genie3 proposed by (Huynh-Thu *et al.* 2010), which utilizes a regression tree based method to infer the GRNs based on expression data (thus cell-type specific). Though, it is worth noting, like in the SpaOTsc case, that CLARIFY is a semi-supervised cell-specific method, we still compare it to a representative cell-type method to gauge the baseline performance.

We use the scMultiSim dataset which has ground truth GRNs. To obtain the Genie3 GRNs, we isolate cells from each cell type (5 total) from the scMultiSim expression data and infer a cell-type GRN for each. Any cell of type *i* will have the same GRN *i*. To obtain the CLARIFY GRNs, we take the block diagonal of the gene-level adjacency matrix $A_{g}$. We compare both CLARIFY and Genie3 to the simulated ground truth using the AUPRC ratio, which allows us to quantify how many folds the candidate model performs better than a random classifier, and has been used in previous work (Pratapa *et al.* 2020). CLARIFY performs better with an AUPRC ratio of 1.48 compared to Genie3’s 1.40 and CeSpGRN’s 1.33—a good result considering CLARIFY’s multiple other functions.

#### 3.3.2 CLARIFY latent gene embeddings indicate valid spatial refinement through global structure while also maintaining local structure information

**Figure 5.**
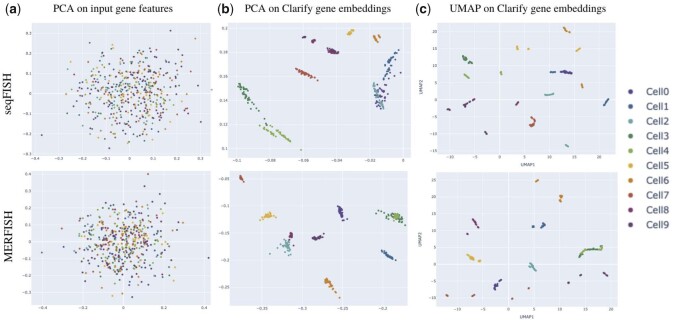
Unsupervised clustering on gene level features/embeddings. (a) PCA on input gene features, (b) PCA on CLARIFY gene embeddings to assess global structure, (c) UMAP on CLARIFY gene embeddings for assessing the local structure.

To assess the spatial refinement of CLARIFY gene embeddings, we used unsupervised clustering. We projected all genes belonging to GRNs of the first 10 cells, across all datasets. Each point in Fig. 5 represents the lower dimensional projection of a gene.

First, to assess the global structure, we compared the projections on the first two Principal Components of the input gene features and the CLARIFY embeddings (Fig. 5a and b, respectively). The input gene features showed virtually no clustering. This was expected because the gene features were constructed on the GRN connected components with Node2Vec. The initial graph consisted of disjoint GRN components, thus no gene from different GRNs were able to share information via the Node2Vec random walks. Hence, the scattered projections across datasets.

However, after embedding the gene features with CLARIFY, we observed a tight clustering of genes belonging to the same cell (Fig. 5c; each cell has a distinct color). Moreover, because PCA preserves global structures (intercluster distance), we also observed that genes of neighboring cells are also clustered. For example, proximal cells Cell0, Cell1, and Cell2 are clustered on the far right of seqFISH plot (b). We also investigated the local structure between CLARIFY Gene embeddings using Uniform Manifold Approximation and Projection (UMAP), which tightly clusters each gene belonging to the same cell and far apart from other genes, showing that local structure is preserved.

Both the PCA and the UMAP plots confirm that CLARIFY gene representations are spatially refined (indicated by the global structure) and cell-specific as well (shown by the UMAP local structure).

Lastly, in order to test if the CLARIFY “refined” GRNs are spatially correlated, we used Spearman correlation again (see Table 3). The baseline experiment was the same as the cell-level heatmap, where each entry represented the euclidean distance between the pair of cell locations, which essentially encapsulates the spatial distribution of the cells. Since each cell is now associated with an adjacency matrix of the corresponding GRN, we therefore tested if the adjacency matrices of each GRN were spatially refined. First, we construct another heatmap/correlation matrix with the same dimensions as the cell by cell analog. Each, *i*, *j* entry represents a “distance” metric between the adjacency matrices corresponding to GRN *i* and GRN *j*. Matrix distance was measured using the Frobenius Norm defined below or alternatively, using the Euclidean distance on the flattened matrix. We calculated each of these pairwise Matrix comparisons and organized them into a heatmap correlation matrix. This was done for both the initial gene adjacencies (inferred by CeSpGRN) and the CLARIFY “refined” gene adjacencies. Finally, analogous to the cell level experiment, we compute the Spearman correlation in two cases: the initial adjacency versus location distribution baseline and the CLARIFY adjacency versus location distribution baseline. In these two cases, we compute the scores either using the entire heatmap matrix or just on the block diagonal. Understandably, there was a lot of sparsity in the entire matrix and the block diagonal entries better represented the cell–cell communities. Across all correlation comparisons along the block diagonal (and both Euclidean and Frobenius distances), there was an increase in correlation with the spatial distribution when using the CLARIFY refined adjacency (block diagonal Correlation coefficient −0.0069 for CeSpGRN versus 0.2079 for CLARIFY refined GRN). For comparisons using the entire matrix, there was a lower increase, which can be explained by the sparsity of data (Correlation coefficient −0.0055 for CeSpGRN versus 0.0766 for CLARIFY refined GRN).

In summary, these results, including (i) unsupervised clustering experiments that indicated both global spatial patterns while maintaining local structure and (ii) the Spearman correlation experiments that quantified increase in spatial correlation after CLARIFY refinement, support our claim that CLARIFY is able to spatially refine gene regulatory networks.

## 4 Conclusion

We present CLARIFY, a graph autoencoder based method that jointly refines both CCIs and cell-specific GRNs. It is the first method that outputs CCIs and GRNs in the same model. The improvements predicted by our tool point to the importance of joint model inference in the future. Our future work will focus on using these regulatory inference tools for problems like the characterization of the tumor microenvironment, or the interplay between tumor cells and immune cells. Since the study of CCIs is still in its infancy, there is much unknown and some common assumptions are needed to be made while designing computational models. Here, we made the assumption that the GRNs of cells which are spatially close are similar. As more knowledge is gained on the spatial landscape of GRNs, the CLARIFY model can be modified to accommodate new information.

## Supplementary Material

btad269_Supplementary_Data
