# Supplementary material for: CLARIFY: cell–cell interaction and gene regulatory network refinement from spatially resolved transcriptomics
Source: Bioinformatics. 2023 Jun 30;39(Suppl 1):i484–93. doi: 10.1093/bioinformatics/btad269 (PMC10311313; doi:10.1093/bioinformatics/btad269)
Supplement: btad269_Supplementary_Data [file btad269_supplementary_data.pdf]

Supplementary Material

Table S1. Table of p-values associated with Fig. 3c for each pairwise comparison per split between CLARIFY and DeepLinc across multiple runs. One-sided Wilcoxon rank sum test was used to confirm that the underlying CLARIFY distributions for each split were stochastically greater than those of DeepLinc.

Test edge ratio	0.1	0.3	0.5	0.7	0.9
seqFISH	4.120e-10	1.476e-10	1.476e-10	1.476e-10	1.544e-10
MERFISH	0.9999	0.8054	0.0152	3.666e-09	1.476e-10
scMultiSim	2.315e-07	1.491e-08	8.745e-05	4.420e-08	4.133e-07

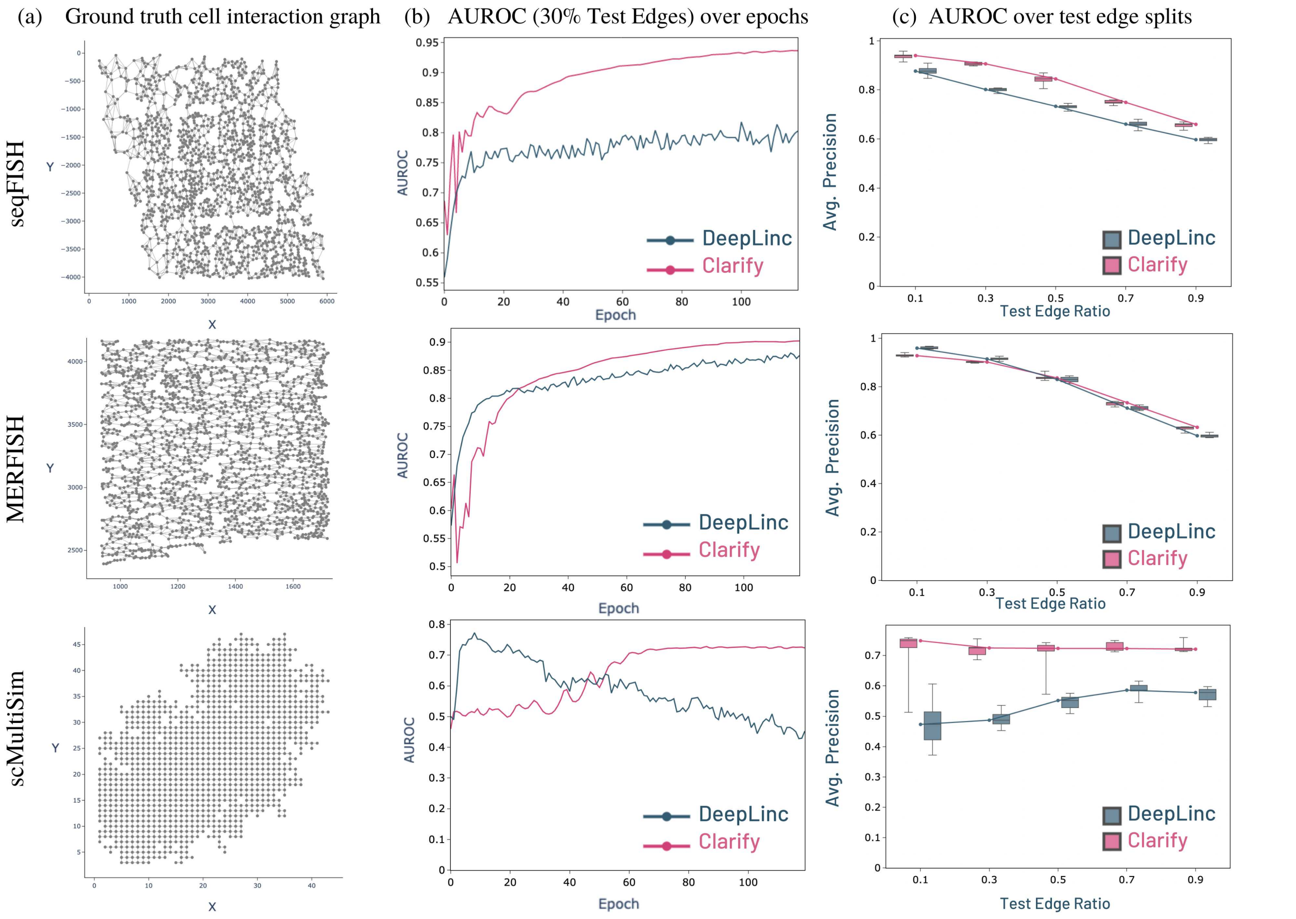

Figure S1. Cell-level experiment performance using AUROC. (a) depiction of the spatial transcriptomics datasets with ground truth cell interaction edges. (b) CLARIFY vs. DeepLinc training AUROC over epochs. (c) CLARIFY vs. DeepLinc AUROC performance over various train/test splits denoted by the % of test edges.

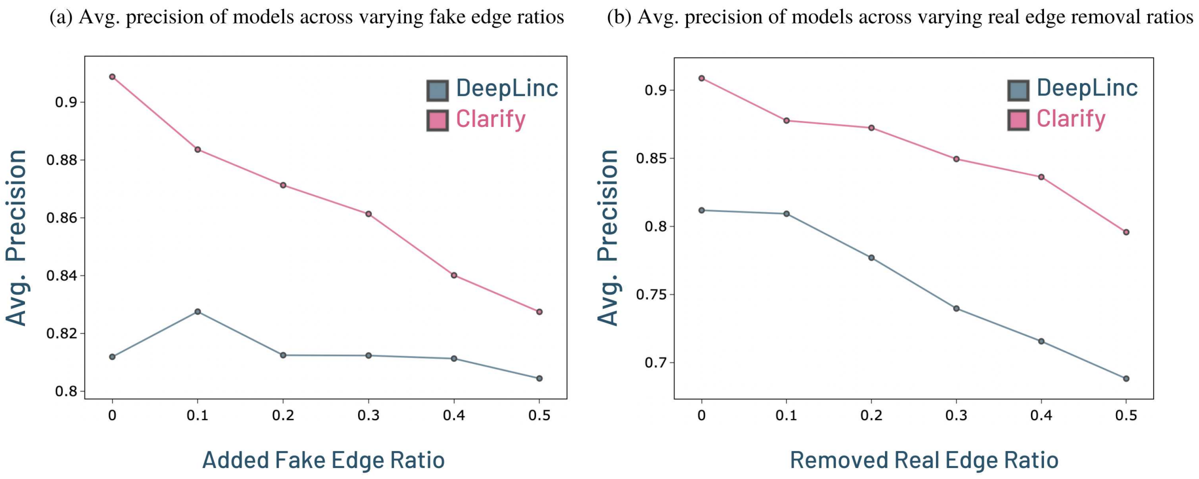

Figure S2. Cell-level experiment with perturbed training set of edges. (a) CLARIFY vs. DeepLinc test set Avg. Precision over different ratios of adding fake edges to the training dataset. (b) CLARIFY vs. DeepLinc test set Avg. Precision over different ratios of removing real edges from the training dataset.
